# Community pharmacy-based hypertension screening: May Measurement Month 2023 in 3 Canadian provinces

**DOI:** 10.1177/17151635241310878

**Published:** 2025-01-24

**Authors:** Stephanie C. Gysel, Tiffany A. Lee, Neil Poulter, Ross T. Tsuyuki

**Affiliations:** Faculty of Medicine and Dentistry, University of Alberta, Edmonton, AB; School of Pharmacy, Memorial University, St. John’s, NL; Imperial College London, London, UK; Faculty of Medicine and Dentistry, University of Alberta, Edmonton, AB

## Introduction

Elevated blood pressure (BP) is the largest contributing risk factor for premature death and disability worldwide.^1^ Approximately 50% of individuals with hypertension are unaware they have the condition; therefore, BP screening is essential. Active clinical screening of asymptomatic patients reduces the lead time between diagnosis and treatment.^1^

May Measurement Month (MMM) is an annual, global BP screening and awareness campaign that launched in 2017. To date, more than 100 countries have participated.^2^ Canadian health professionals have previously participated in MMM; however, it has never been conducted in community pharmacies. Pharmacies have the advantage of being highly accessible to the public, and pharmacy personnel are well-positioned to engage in BP screening. We conducted a BP screening event in community pharmacies in Newfoundland and Labrador (NL) on World Hypertension Day in 2022, which helped us to scale-up for MMM23 in 3 provinces.^3^ In this article, we provide a brief research report on the MMM23 campaign in Canadian community pharmacies.

## Methods

### Study design and setting

A cross-sectional study of consenting adults was conducted in Alberta (AB) and British Columbia (BC) (22 pharmacies) from May 1 to July 31, 2023, and in NL (30 pharmacies) from May 17 to 31, 2023. All research sites followed a common protocol, available on the MMM website (www.maymeasure.org).^4^ In addition, pharmacy personnel completed mandatory training, including a virtual short course on the proper technique for automated blood pressure measurement (https://campus.paho.org/en/course/virtual-course-accurate-automated-blood-pressure-measurement-2020)^5^ plus 2 hours of training with the research team. Each study site was encouraged to engage pharmacy technicians and pharmacy students in measurement of BP and consenting procedures. Pharmacy personnel were permitted to conduct BP screening via appointment or scheduled screening days based on their preference.

### Sampling and recruitment

All consenting adults (≥18 years) were eligible for inclusion. There were no exclusion criteria. Convenience sampling was most appropriate as it aligned with the goals of MMM to facilitate opportunistic screening of BP. Participants were recruited through community-based billboard signage, radio media advertisements, and social media modalities. Study promotional materials were also distributed by pharmacy research sites. The desired sample size for this project was determined to be 592, calculated using the equation for frequency in a population with a population size of 7,000,000 adults, a prevalence of elevated BP at 25%, and a 5% margin of error.

### Data collection

Participant self-reported data included demographic information (e.g., age, sex, ethnicity), medical history, use of antihypertensives, and lifestyle information. Pharmacy personnel collected 3 blood pressure and pulse readings from each participant using an automated device. BP readings were taken with a 1-minute interval between readings using an appropriately sized BP cuff for the participant’s arm and after the participant had been seated for 5 minutes. Automated office BP devices recommended by Hypertension Canada’s Device Recommendation Program (www.hypertension.ca/healthcare-professionals/recommended-devices)^6^ were provided to each community pharmacy by the research team. Data were entered in the study database (using the bespoke MMM App in AB and BC and REDCap in NL), including BP measurements.

### Outcomes

The primary outcome was elevated BP, defined as BP ≥140/90 mmHg or BP ≥130/80 mmHg for individuals with diabetes. The mean of the second and third readings was used to estimate BP. Secondary outcomes included the proportion of elevated BP in participants with known hypertension, no history of hypertension or diabetes, and in females.

Participants with elevated BP were provided with written dietary and lifestyle advice along with their BP record. Pharmacist intervention was not a part of the MMM23 protocol; however, pharmacists provided participants who had elevated BP advice on management and follow-up based on provincial scope of practice.

### Data analysis

Analysis was performed using SAS 9.4 software. Descriptive statistics were used to examine participants’ demographic information and clinical characteristics, including frequency counts and percentages for categorical variables, and mean and standard deviation (SD) for continuous variables. Only participants with 3 BP readings documented in the study database were included in the analysis of BP results.

## Results

A total of 736 participants took part in the Canadian community pharmacy MMM23 project. Most participants (*n* = 482, 66%) were female, and the mean age was 52 (SD 18.6) years. In terms of medical history, 34.2% (*n* = 252) of participants had a history of hypertension and 14.0% (*n* = 103) had a history of diabetes. Further details regarding participant characteristics are provided in Table 1.

**Table 1:** Participant baseline characteristics

Characteristic	Frequency (*N* = 736)
Mean (SD) age at screening, years	52 (18.6)
Female gender, *n* (%)	482 (65.5)
Diabetes, *n* (%)	103 (14.0)
Known hypertension, *n* (%)	252 (34.2)
Current smoker, *n* (%)*	73/671 (10.9)

* Percentage was calculated based on available data: 195 participants in Alberta and 92 participants in British Columbia. SD, standard deviation.

A total of 733 participants (99.6%) had 3 BP readings documented in the database and were included in the analysis of BP (see Table 2). Overall, the mean of the last 2 measurements (i.e., readings 2 and 3) for systolic BP was 123.8 mmHg (SD 15.6) and for diastolic BP was 73.5 mmHg (SD 10.3).

**Table 2:** Elevated BP and subgroups of interest with elevated BP

Subgroup of interest	Frequency, *n*/*N* (%)
Elevated BP*	152/733 (20.7)
Known hypertension^†^	88/251 (35.1)
No history of hypertension^‡^	64/482 (13.3)
Diabetes^§^	53/103 (51.5)
Female**	96/480 (20.0)

BP, blood pressure; *n*, number of subjects with characteristic of interest; *N*, total number of subjects.

* Participants with elevated BP.

† Participants with elevated BP and known hypertension.

‡ Participants with elevated BP and no history of hypertension.

§ Participants with elevated BP and diabetes.

** Female participants with elevated BP.

## Discussion

The World Health Organization recommends that every adult be screened annually for elevated BP through opportunistic and accessible screening programs.^1^ Community pharmacies are ideal locations to conduct opportunistic screening of BP. We leveraged the unique front-line position of pharmacy personnel to engage with the annual MMM campaign. In doing so, we were able to reach many individuals across 3 provinces and identified more than 150 individuals (21% of the study population) with elevated BP.

The findings of our project are in line with current Canadian statistics, which report that 1 in 5 Canadians have elevated BP.^7^ In contrast, the 2019 International MMM data revealed a prevalence of 34% elevated BP; however, comparisons are difficult due to the heterogeneity of sampling methods.^8^ In our study, elevated BP was observed among a high proportion of participants with known hypertension (35.1%) and diabetes (51.5%); these findings exceed Canadian estimates.^7^ It should be acknowledged that these findings may be the result of reduced access to in-person care during the pandemic^9^ and the ongoing shortage of family physicians across the country.^10^

Our study has several strengths. We were able to reach more than 750 individuals across 3 provinces and raise awareness about the importance of BP measurement. In addition, we provided members of the public with timely BP measurement and assessment by highly qualified health professionals. The naturalistic design of this project allows for individualized integration in community pharmacy workflows.

Our study also has limitations. First, self-selection bias likely occurred, as those individuals who are most interested in their cardiovascular health may have presented for screening. This may have led to lower rates of elevated BP due to health-conscious behaviours of participants or, conversely, higher rates of elevated BP as these participants elected to be screened. Second, it should be acknowledged that hypertension is diagnosed and monitored using measurements on more than a single occasion. As such, we can only report “elevated BP” rather than true hypertension. That said, we were able to effectively identify 150 individuals with elevated BP who required follow-up and further assessment of their BP, and there were undoubtedly improved health outcomes for these participants. Finally, our limited and non-random sampling does not allow for a robust assessment of prevalence; however, our results are similar to prevalence results previously reported.^7^

In completing this project, we have been able to identify the strengths and limitations of community pharmacy-based BP screening. Pharmacy personnel acceptability of this initiative will be discussed in a subsequent article. Future research should focus on studying the implementation of a pan-Canadian community pharmacy-based BP screening and awareness campaign in conjunction with Hypertension Canada. We will also evaluate patient acceptability of BP screening in community pharmacies.

## Conclusion

Identifying individuals with elevated BP is the starting point for better BP management. By engaging with the global MMM campaign, we were able to expand hypertension screening to community pharmacies to screen more than 700 individuals across 3 Canadian provinces. Further, we identified more than 150 individuals with elevated BP. Community pharmacies are highly accessible and serve as important health care hubs for individuals seeking access to BP screening. Our experiences from MMM23 will guide the future scale-up of a national community pharmacy-based screening program.
